# Histone methyltransferase SET8 is regulated by miR-192/215 and induces oncogene-induced senescence via p53-dependent DNA damage in human gastric carcinoma cells

**DOI:** 10.1038/s41419-020-03130-4

**Published:** 2020-10-30

**Authors:** Xiaojing Zhang, Yin Peng, Yuan Yuan, Yuli Gao, Fan Hu, Jian Wang, Xiaohui Zhu, Xianling Feng, Yulan Cheng, Yanjie Wei, Xinmin Fan, Yaohong Xie, Yansi Lv, Hassan Ashktorab, Duane Smoot, Song Li, Stephen J. Meltzer, Gangqiang Hou, Zhe Jin

**Affiliations:** 1 Guangdong Key Laboratory for Genome Stability & Disease Prevention and Regional Immunity and Diseases, Department of Pathology, Shenzhen University School of Medicine, Shenzhen, Guangdong 518060 People’s Republic of China; 2 Department of Medicine/GI Division, Johns Hopkins University School of Medicine and Sidney Ki-mmel Comprehensive Cancer Center, Baltimore, MD 21287 USA; 3 Center for High Performance Computing, Shenzhen Institutes of Advanced Technology, Shenzhen, Guangdong 518000 People’s Republic of China; 4 Department of Medicine and Cancer Center, Howard University College of Medicine, Washington, DC 20060 USA; 5 Department of Medicine, Meharry Medical Center, Nashville, TN 37208 USA; 6 Shenzhen Science & Technology Development Exchange Center, Shenzhen Science and Technology Building, Shenzhen, Guangdong 518055 People’s Republic of China; 7 Department of Medical Image Center, Kangning Hospital, Shenzhen, Guangdong Province 518000 People’s Republic of China

**Keywords:** Cancer microenvironment, Diagnostic markers

## Abstract

Gastric cancer (GC) is the most common cancer throughout the world. Despite advances of the treatments, detailed oncogenic mechanisms are largely unknown. In our previous study, we investigated microRNA (miR) expression profiles in human GC using miR microarrays. We found miR-192/215 were upregulated in GC tissues. Then gene microarray was implemented to discover the targets of miR-192/215. We compared the expression profile of BGC823 cells transfected with miR-192/215 inhibitors, and HFE145 cells transfected with miR-192/-215 mimics, respectively. SET8 was identified as a proposed target based on the expression change of more than twofold. SET8 belongs to the SET domain-containing methyltransferase family and specifically catalyzes monomethylation of H4K20me. It is involved in diverse functions in tumorigenesis and metastasis. Therefore, we focused on the contributions of miR-192/215/SET8 axis to the development of GC. In this study, we observe that functionally, SET8 regulated by miR-192/215 is involved in GC-related biological activities. SET8 is also found to trigger oncogene-induced senescence (OIS) in GC in vivo and in vitro, which is dependent on the DDR (DNA damage response) and p53. Our findings reveal that SET8 functions as a negative regulator of metastasis via the OIS-signaling pathway. Taken together, we investigated the functional significance, molecular mechanisms, and clinical impact of miR-192/215/SET8/p53 in GC.

## Introduction

Gastric cancer (GC) is one of the most common cancers all over the world, especially in Asia. Despite the improved surgical and adjuvant treatment approaches^1^, the prognosis of GC patients is still poor. Five-year overall survival of GC is generally 25–30%. Therefore, it is necessary to better understand the molecular pathogenesis of GC. In this study, we focus on the molecular mechanism of GC development and the discovery of the novel, dependable, and noninvasive biomarkers for GC.

microRNA (miR) is a small, single-stranded RNA, and negatively regulates the target genes by interacting with the untranslated regions (UTRs) of targets^2^. It plays a critical role in cancer-related proliferation, migration, apoptosis, and cell-cycle progression. In our previous study, we investigated the miR expression profiles in human GC using miR microarrays. We found miR-192/215 were upregulated in GC tissues^1,2^. MiR-192/215 belongs to the miR-192 family, possessing the same octameric seed sequence. In this study, we further confirmed that miR-192/215 function as oncogenic miRs (oncomiRs) in GC. To discover targets of miR-192/215 that were significantly dysregulated in GC, custom microarray analyses with an expanded set of probes were performed. HFE145 and BGC823 cells were transfected with mimics or inhibitors of miR-192 or -215 or a negative control (NC) miR. Genes exhibiting more than twofold changes in two comparisons were regarded as the proposed targets. SET8 was properly identified by these filtering criteria.

Histone methyltransferase SET8 (also known as SETD8, PR-SET7, and KMT5A) belongs to the SET domain-containing methyltransferase family and specifically catalyzes monomethylation of H4K20me, that is, the formation of H4K20me1^3^. SET8 exhibits diverse functions in transcriptional regulation, DNA repair, tumor metastasis, genome integrity, and cell-cycle progression^4^. SET8 has been proposed to be associated with cancer occurrence and progression by affecting cell proliferation^5^ and promoting the epithelial–mesenchymal transition (EMT)^4,6^ in osteosarcoma and breast cancer. Interestingly, SET8 has also shown involvement in the oncogene-induced senescence (OIS) mechanism, a tumor-suppressing response that must be disrupted for cancer to develop. Our results suggest that SET8 is directly regulated by miR-192/215 in human GC, as demonstrated by its decreased mRNA and protein expression in GC. Binding of miR-192/215 to the 3′UTR of SET8 resulted in the promotion of GC cell proliferation, migration, and metastasis. In addition, SET8 was shown to be involved in triggering OIS in GC in vivo and in vitro, which was activated by p53 in GC. The functional role of the miR-192/215-SET8-p53 axis in repressing the senescence signal pathway exacerbates the progression of GC.

## Methods

### Cell lines, patient samples, culture conditions, and reagents

Immortalized human normal gastric epithelial cells (HFE145) were obtained from Drs. Hassan Ashktorab and Duane Smoot. BGC823, SGC7901, and AGS were obtained from Cell Bank of the Chinese Academy of Sciences (Shanghai, China). MKN28 cells were obtained from the American Type Culture Collection and China Infrastructure of Cell Line Resources, respectively. The cell lines were freshly authenticated in last year. All cells were grown in DMEM media supplemented with 10% fetal bovine serum (FBS) and 1% penicillin/streptomycin (P/S) at 37 °C in 5% CO_2_ incubator. Fresh GC samples were obtained from the first Affiliated Hospital of Shenzhen University, and patients prior to radiotherapy and chemotherapy. In this study, 48 patients were collected and histologically confirmed new cases of GC. All the processes were consistent with the requirement of the Institutional Review Board of The School Medicine of Shenzhen University (No. 201505001). CDDP (Cis-diamminedichloroplatinum, best known as cisplatin, CDDP) was purchased from Calbiochem (Billerica, MA). To induce DNA damage, cells were treated with CDDP at indicated concentrations and harvested for protein isolation at the stated time points.

### Gene microarray

Gene microarrays were carried out to identify the targets of miR-192/215, all of which were according to the previously described^2^.

Briefly, gene microarrays were carried out on the Agilent Whole Genome Oligo Microarrays (4x44K, Agilent, Santa Clara, CA, USA) in GC cells. Microarrays performance and analysis were performed on two groups of cell lines: BGC823 cells with miR-192 and -215 inhibitors, and HFE145 cells with miR-192 and -215 mimics. Twofold differences in gene expression determined genes considered for further analysis. Data were extracted and further analysis was conducted by Agilent Gene Spring GX 11.5.1 software.

### Transfection of miRs and siRNAs

Cells were seeded at 50% confluence, and miRs, inhibitors or mimics with concentration of 60 nM were transfected via Lipofectamine RNAi MAX (Invitrogen). Synthesized RNA Mimics or inhibitors of miRs were purchased from Dharmacon (Lafayette, CO, USA). SET8siRNA (SET8si) and miRs antagomir with cholesterol-conjugated used for assays in vivo were synthesized by Ribobio (Guangzhou, China). SiRNAs were mixed to achieve the necessary knockdown of SET8 protein expression as determined in Fig. 3A. Therefore, the mixed siRNAs were used in the needed experiments. Nonspecific controls for mimics, inhibitors, and siRNAs were used as negative controls (NC). All the SET8si sequences are listed in Table S1. In order to detect the expression of senescence-associated secretory phenotype (SASP), both RNA and protein were harvested after 48 and 96 h of transfections. RNA was extracted as described in the qRT-PCR section. Protein extraction was followed with the previously described in the western blot section^2^.

### Real-time qRT-PCR

Total RNA, including miRs from the tissue samples and cultured cells, was extracted using TRIzol reagent (Invitrogen, USA) according to established protocols. The expression level of miRs was analyzed by quantitative real-time PCR (qRT-PCR) using the All-in-One miRNA qRT-PCR Detection Kit (Gene Copoeia). The Advantage RT-for-PCR Kit (Invitrogen, USA) was used to generate cDNA for the detection of mRNA. The expression level of SET8, IL-1a, IL-1b, IL-6, IL-8, p53, and p21 were analyzed by qRT-PCR (Takara, Dalian). GAPDH and U6 were used as the inner reference to normalize the mRNA and miRs expression, respectively. RT-PCR was carried out to detect the levels of EMT associated genes (E-cadherin, Snail, MMP9, Vimentin, and ZEB1) regulated by SET8. Subcutaneous tumor tissues were examined with SET8, p21, and p53 to test the proliferation abilities and the switch of OIS with different treatments. The relative mRNA or miRs levels were calculated using the comparative Ct method (2^−ΔΔCt^). Primers were indicated in supplementary Table 1.

### Tissue microarrays and IHC

Tissue microarrays, paraffin-embedded GC samples were collected from 90 patients who underwent GC resection without prior radiotherapy and chemotherapy including GC and para-cancer tissues. All the cases were attached with complete clinical data for further analysis. Immunohistochemistry (IHC) was performed according to the protocol to examine the expression of SET8 in GC tissues. EnVision + detection system (Dako) was used following the manufacturer’s instructions. In brief, sections overnight incubated at 4 °C with a 1:500 dilution of monoclonal anti-SET8 (ab3798, Abcam) and a 1:5000 dilution of the second antibody. All the clinical pathology data were collected and the correlations between SET8 and the clinical data were analyzed. Positive score of SET8 was calculated according to the percent positivity of stained cells (0–4 scores, 3–4 regarded as the high expression) and the staining intensity (0–3, 0 means negatively stained, 3 means strongly stained). The value *p* < 0.05 was regarded as the significant difference.

### Kaplan–Meier survival analysis

In order to analyze the survival significance of SET8, Kaplan–Meier survival analysis was performed. Data of GC patients were from TCGA database and our tissue microarray. TCGA has so far collected more than 11,000 cancer patient specimens and matched normal tissues. At present, it is the largest and richest cancer genome database in the world. In our analysis, there were 415 cases of stomach adenocarcinoma (STAD) in total, but only 408 clinical documents of patients were collected. Therefore, 408 cases with STAD were involved in the survival analysis of SET8. In TCGA database, we also analyzed the survival significance of miR-192. As seven patients with missing survival time, 389 cases were analyzed. Finally, 90 GC cases of tissue microarrays were executed the Kaplan–Meier survival analysis as well.

### Cell lysis, SDS-PAGE, and immune blot

Cells were lysed with RIPA lysis buffer (50 mM Tris, pH 8.0; 150 mM NaCl, 1% NP-40; 0.5% sodium deoxycolate; 0.1% SDS; 1 mM Benzamidin-HCl; 0.5 μg/ml Aprotinin), and equal amounts of protein were electro-phoretically separated in a polyacrylamide 8–12% gel. The following primary antibodies were used: SET8 (1:1000), p53 (1:1000), and p21 (1:1000) were all purchased from Cell Signal Technology, USA. Senescence Marker Antibody Kit (CST, USA), including senescence-associated cell-cycle arrest (p16 INK4A, p21 Waf1/Cip1), senescence-associated DNA damage (Phospho-gamma-Histone H2A.X,γH2A.X), and the SASP (HMGB1, IL-6, TNF-alpha, MMP3) were used to detect the changes of senescence phenotype. DNA damage checkpoint proteins, Chk1 (2G1D5), Phospho-Chk1 (Ser345) (133D3), Chk2 (1C12), and Phospho-Chk2 (Thr68) (C13C1) (CST, USA) were also measured. GAPDH (1:2000) was used as the inner control. All the procedures were following the previous details^7^.

### Luciferase activity assay

Sequences of 3′-UTR of SET8 containing wild type (wt) or mutant (mut) were synthesized by Gene copoeia (Guangzhou, China) and cloned into the luciferase reporter vector pEZX-MT06 to acquire the SET8-3′-UTR reporter constructs (pEZX-wt-SET8 and pEZX-nut-SET8). Co-transfection of miRs and luciferase report vectors, miRs of 60 nM, and plasmids of 40 ng were transfected using Lipofectamine 3000 according to the instructions (Invitrogen, USA). The luciferase activity was measured 48 h after transfections by the Dual-Luciferase Reporter Assay Kit (Promega)^7^. Each assay was repeated in three independent experiments.

### Cell proliferation, migration, and apoptosis assays

For the assays in vitro, we detected the change of bio-behavioral functions of GC cells transfected with SET8si or siRNA NC. The cell proliferation and cell migration assays of transfected GC cells were determined as previously described^2,7^. EdU proliferation assay (RiboBio Inc.) was carried out according to the manufacturer’s instructions. HFE145 and SGC7901 cells were plated in 6 cm plates with different transfections, and the cells incubated with 50 μM EdU for 5 h. The images were taken by confocal microscope.

A wound-healing assay was performed to measure cell migration. Briefly, cells were plated in triplicate in a 6-well plate. Linear scratch wounds were created by 200 μl sterile pipette tip when each well was confluent cells. Artificial scratch was made and wound images were photographed and migration was monitored at 0, 24, 48 h after scratching using Olympus 1X71 camera system. Each experiment was repeated three times.

The extent of apoptosis was monitored by using the Annexin-V FLUOS Staining Kit (Invitrogen, USA), following the manufacturer’s instructions. In short, cells were collected and re-suspended in 100 ml of solution with Annexin-V-FLUOS labeling and PI-staining solution (Roche Applied Science, Indianapolis, USA). Then cells incubated at room temperature for 15 min in the dark, analyzed by the flow cytometry using a FACSCA instrument with CELLQUEST analysis software (BD Biosciences). All these experiments repeated three times.

### Cell counting kit-8 assay

The cells were seeded in 96-well plates (2.5 × 10^3^ cells/well) with 100 μl cell suspension in each well and incubated for 7 days. Cells were treated with CDDP at 0, 5, 10, and 20 μM concentrations. CCK-8 assay was performed by adding CCK-8 for 2 h according to the manufacture’s instruction. The absorbance value of each well was measured with a microplate reader of 450 nm. Each experiment was repeated three times.

### Animal models

To evaluate the tumor growth in vivo, BALB/c-nu female nude mice (4–5 weeks old) were purchased from the Guangdong Province Laboratory Animal Center (Guangzhou, China). The mice were bred in specific pathogen-free conditions in mesh cages under controlled conditions of temperature (23 ± 3°C) and relative humidity (50 ± 20%). Animal models were set up followed the procedure as previously described^2^. Briefly, 1 × 10^6^ BGC823 cells with or without cholesterol-conjugated negative control (NC) or miR-192 inhibitors (192inh) or SET8siRNA (SET8si) were suspended in 100 ml PBS and injected s.c. into the opposite rear flank of the nude mice. Each group included five mice. After 5 days, 10 nmol cholesterol-conjugated inhibitors or siRNAs were injected for each implant for 3 days. Then treatments were delivered once a week until sacrificed. Tumor size was measured by caliper measurement of two perpendicular diameters of the implants. Tumor-bearing mice were sacrificed on day 21. All the tumor tissues were fixed, paraffin-embedding, and sectioned. To evaluate metastasis in vivo, 1 × 10^6^ cells were injected into the tail veins of mice (*n* = 5 per group). All procedures were performed as previously described^2^. Shortly, mice were euthanized after 8 weeks of injections, and the lungs were removed, rinsed, fixed, and subjected to pathological examination. Tissues were stained by H&E, and metastasis was quantified by counting tumor lesions under a microscope. All protocols for animal studies were reviewed and approved by the Institutional Animal Care and Use Committee of Medical College of Shenzhen University. A method of randomization was used in the animal models. Animal models were blinded to the group allocation during the experiment.

### SA-β-Gal staining

Cells were fixed in 2% formaldehyde and 0.2% glutaraldehyde in PBS for 5 min at room temperature. Then cells were stained for the SA-β-Gal according to the manufacturer’s instructions (Senescence β-Galactosidase Staining Kit, Beyotime, China). SA-β-Gal cells and total nuclei were counted manually, and calculated the positive ratio of the SA-β-Gal-positive cells.

### Statistical analysis

Statistical analysis was performed using Microsoft Excel. The analysis was performed using paired Student’s *t*-tests. The results are presented as the mean ± SD (standard deviation, SD). The correlation test was evaluated with *χ*^2^ test. One-way ANOVA was used to test the significance of assays in vitro.

## Results

### SET8 expression was low in GC tissues and cells

**Fig. 1:**
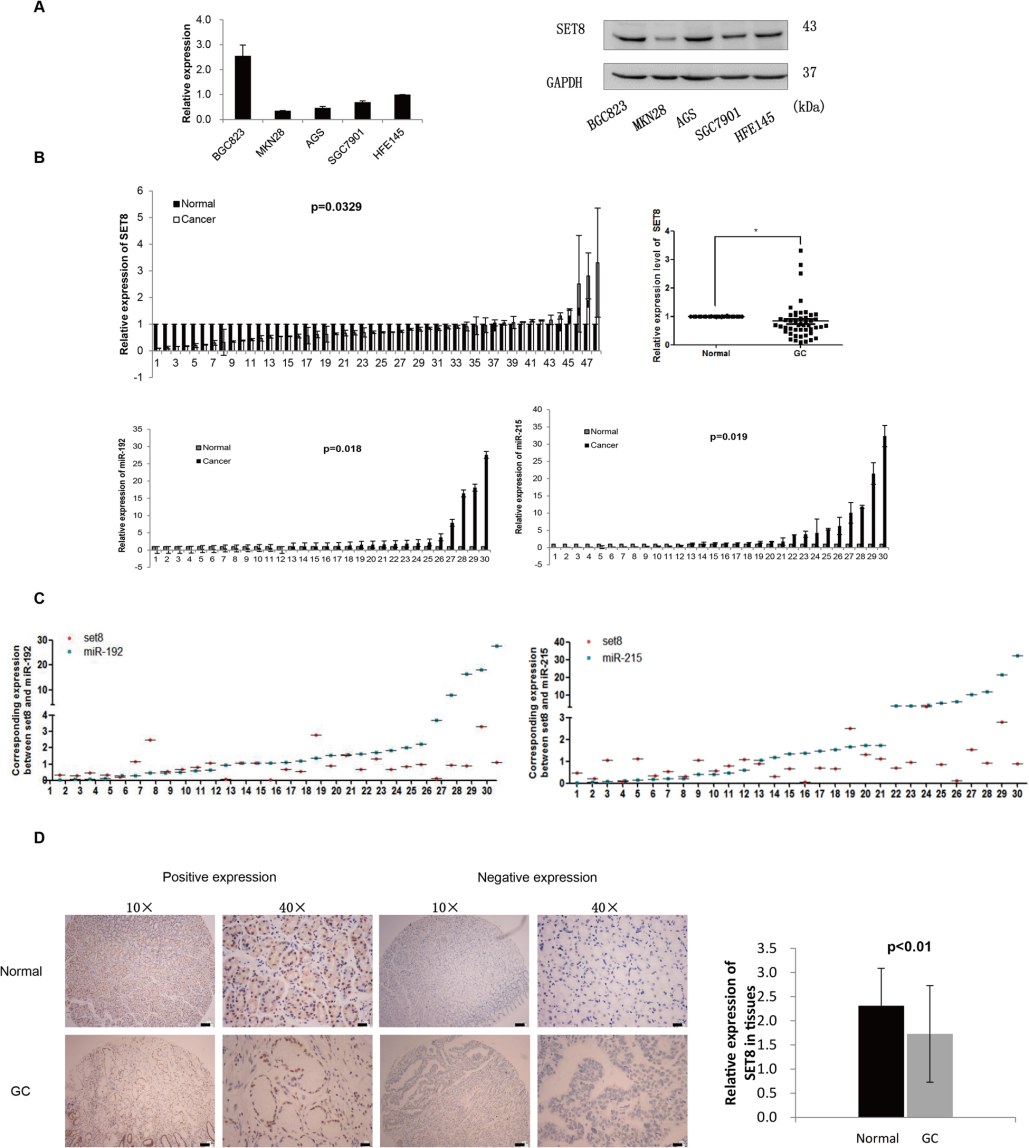
Expression levels and survival analysis of SET8 in GC. **A** mRNA and protein levels of SET8 in five cancer cell lines (BGC823, MKN28, AGS, SGC7901, and HFE145). **B** Up: qRT-PCR analysis of SET8 in 48 pairs of GC tissues. The mRNA levels relative to a normal control were normalized to 1. Down: expression of miR-192/215 in 30 paired GC tissues. **C** Scatter plot of SET8 and miR-192/215 corresponding expression. **D** IHC was performed to determine the protein levels of SET8 in a GC-tissue microarray.

To determine the expression level of SET8 in GC, we detected the mRNA and protein level of SET8 in gastric cells and tissue. Result showed SET8 mRNA and protein were found to be reduced in three GC cell lines (SGC7901, AGS, and MKN28), but not in HFE145 cells (Fig. 1A). Expression levels of SET8 in 48 paired fresh GC tissues were significantly lower in many GC tissues than in normal mucosa (*p* < 0.05). Compared with normal tissues, only 12/48 pairs showed over-expression of SET8 in cancerous tissues, while 36/48 tissue pairs showed under-expression of SET8 in GC (Fig. 1B up).

To analyze the correlation between the expression of miR-192/215 and SET8, we also measured the expression of miR-192/215 in 30 of these 48 paired tissues. Correlation analyses between miR-192/215 and SET8 indicated that miR-192 and -215 were highly expressed in GC tissues, a finding that was consistent with our previous results^1,2^ (Fig. 1B down). However, there was no statistically significant difference in the correlation between the miR-192/215 and SET8 levels (Fig. 1C & Figure S1). Therefore, SET8 was found to be expressed at low levels in GC tissues and cells, which suggests that it may function as a suppressor of GC.

A GC-tissue microarray comprising 180 GC samples of primary tumors and normal tissues was used to detect SET8 protein levels. SET8 protein levels were significantly lower in cancer tissues when compared with normal tissues (Fig. 1D). Moreover, scores from IHC staining showed that 81 of the 90 samples exhibited high expression of SET8 in normal mucosa. All patients who supplied samples for the GC-tissue microarray were followed up for 5 or 6 years, and their complete clinical-pathological data were collected for further analysis. Significant correlations were revealed between the SET8 expression levels and the AJCC’s stage and the extent of remote metastasis (Table 1). Thus, SET8 expression was downregulated in the latter stage of AJCC (stages 3 and 4), relative to the expression level in the early stage of AJCCs (stages 1 and 2). Furthermore, the expression of SET8 was significantly reduced in remote metastases. We therefore speculate that decreased expression of SET8 may play a key role in the invasion and metastasis of GC. Furthermore, we executed survival analyses of miR-192 and SET8 using the survival data from TCGA (The Cancer Genome Atlas, TCGA) and our tissue microarray. Unfortunately, these analyses failed to demonstrate statistical differences in patient survival probabilities attributable to miR-192 or SET8 (Figure S2).

**Table 1:** Relationship between SET8 expressions and clinicopathologic features in GC.

Features	*N*	High expression	Low expression	*P*	*χ*^2^
All case	90	67	23		
90					
*Age*					
<55	25	18	7	0.742	0.109
≥55	65	49	16		
*Gender*					
Male	53	41	12	0.448	0.575
Female	37	26	11		
*Tumor size*					
<5 cm	34	28	6	0.18	1.796
≥5 cm	56	39	17		
*Differentiation*					
Well-moderate	21	18	3	0.176	1.829
Poor	69	49	20		
*Serosal invasion*					
No	9	9	0	0.064	3.433
Yes	81	58	23		
*Vessel invasion*					
No	61	48	13	0.181	1.792
Yes	29	19	10		
*Lymphatic metastasis*					
No	23	19	4	0.198	1.082
Yes	67	48	19		
*AJCC’s stage*					
1 + 2	36	31	5	*0.038*	4.293
3 + 4	54	36	18		
*Remote metastasis*					
No	86	66	20	*0.02*	5.379
Yes	4	1	3		

Italic values indicate statistical significance *p* < 0.05.

### SET8 inhibits proliferation and migration in GC in vitro and in vivo

**Fig. 2:**
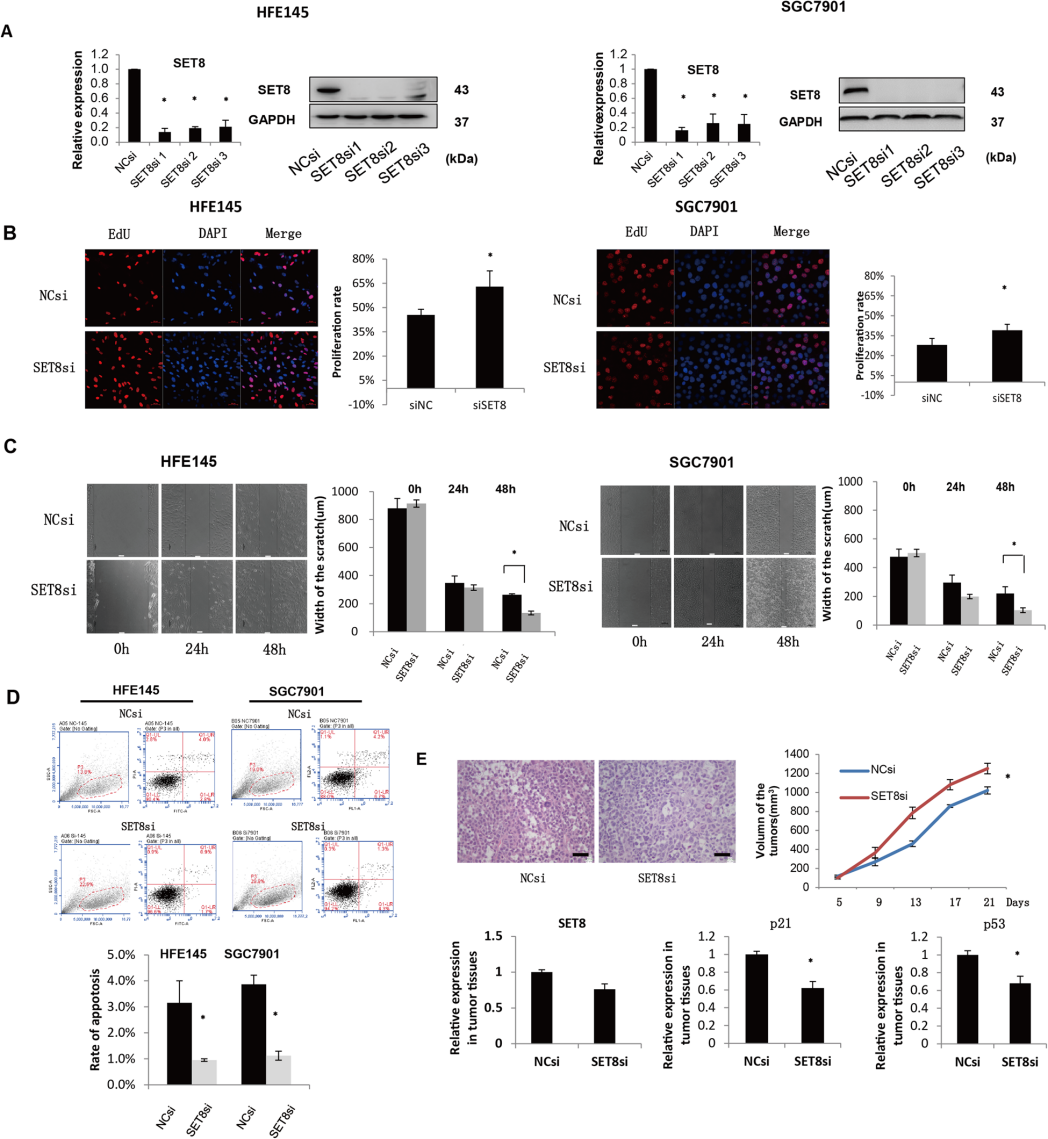
SET8 functions as a negative regulator in the proliferation and migration of GC in vitro and in vivo. **A** Transfection efficiency of SET8si. There were three strips of SET8sis. Cells were transfected with SET8si, and expression of SET8 was thereby almost deleted, especially with respect to protein levels. **B**–**D** Functional in vitro assays of SET8. EdU assays (**B**) showed that the inhibition of SET8 clearly promoted the proliferation of both HFE145 and SGC7901 cells. Results of the scratch test (**C**) indicated that compared with NC siRNA, knockdown of SET8 significantly promoted the migration of gastric cells after transfection for 48 h. Apoptosis (**D**) was determined by flow cytometry using annexin-V-PI staining. SET8 induced apoptosis in both HFE145 and SGC7901 cells. **E** Functional in vivo assays of SET8. Subcutaneous tumor assays suggested that lower expression of SET8 facilitated the growth of GC cells in vivo. In these tissues, SET8 was decreased by SET8si as effectively as it was in vivo. In the subcutaneous tumor tissues, p53 and p21 were regulated by SET8. *P < 0.05. 192, miR-192; 215, miR-215; inh, inhibitor; si, siRNA.

In vitro assays were conducted to identify the biological behavior of SET8 in GC. The efficiency of SET8si was validated by qRT-PCR and protein blot (Fig. 2A). EdU proliferation assays showed that inhibition of SET8 led to significant promotion of proliferation of SGC7901 and HFE145 cells relative to the control (Fig. 2B, *p* < 0.05). Wound-healing assays were performed to examine the invasive ability of SET8 in GC. These showed that monolayers of SGC7901 and HFE145 cells that were transfected with SET8si displayed increased cell invasion over 48 h, as their scratch gaps were closer than those of NC cells (Fig. 2C, *p* < 0.05).

Meanwhile, emerging data have shown that SET8 regulates carcinogenesis by apoptosis^8^. Consequently, Cells treated with SETsi showed an inhibition of apoptosis compared to controls as determined by flow cytometry detection of Annexin-V and propidium iodide (PI) positive cells (Fig. 2D, *p* < 0.05). In HFE145 cells, the apoptosis rate was also decreased by inhibition of SET8. These findings confirm that SET8 is closely involved in cell proliferation, migration, and apoptosis in GC.

To confirm the proliferation-suppression role of SET8 in vivo, we preformed subcutaneous tumor xenograft assays. This showed that inhibition of SET8 led to distinctly larger tumors in the treated group than in the control group. Thus, in summary, we showed that SET8 played a crucial role in the promotion of GC. The p53 and p21 expression were decreased after the level of SET8 was reduced (Fig. 2E). In addition, we also examined the expression of EMT markers regulated by the change of SET8 in vivo. Results showed that, compared with the siRNA control group, expression of Snail, MMP9, Vimentin, and ZEB1 was increased in the tissues treated with SET8si, and E-cadherin was expressed at low level (Figure S3).

### P53 and p21 are involved in the OIS induced by SET8 in GC cell

**Fig. 3:**
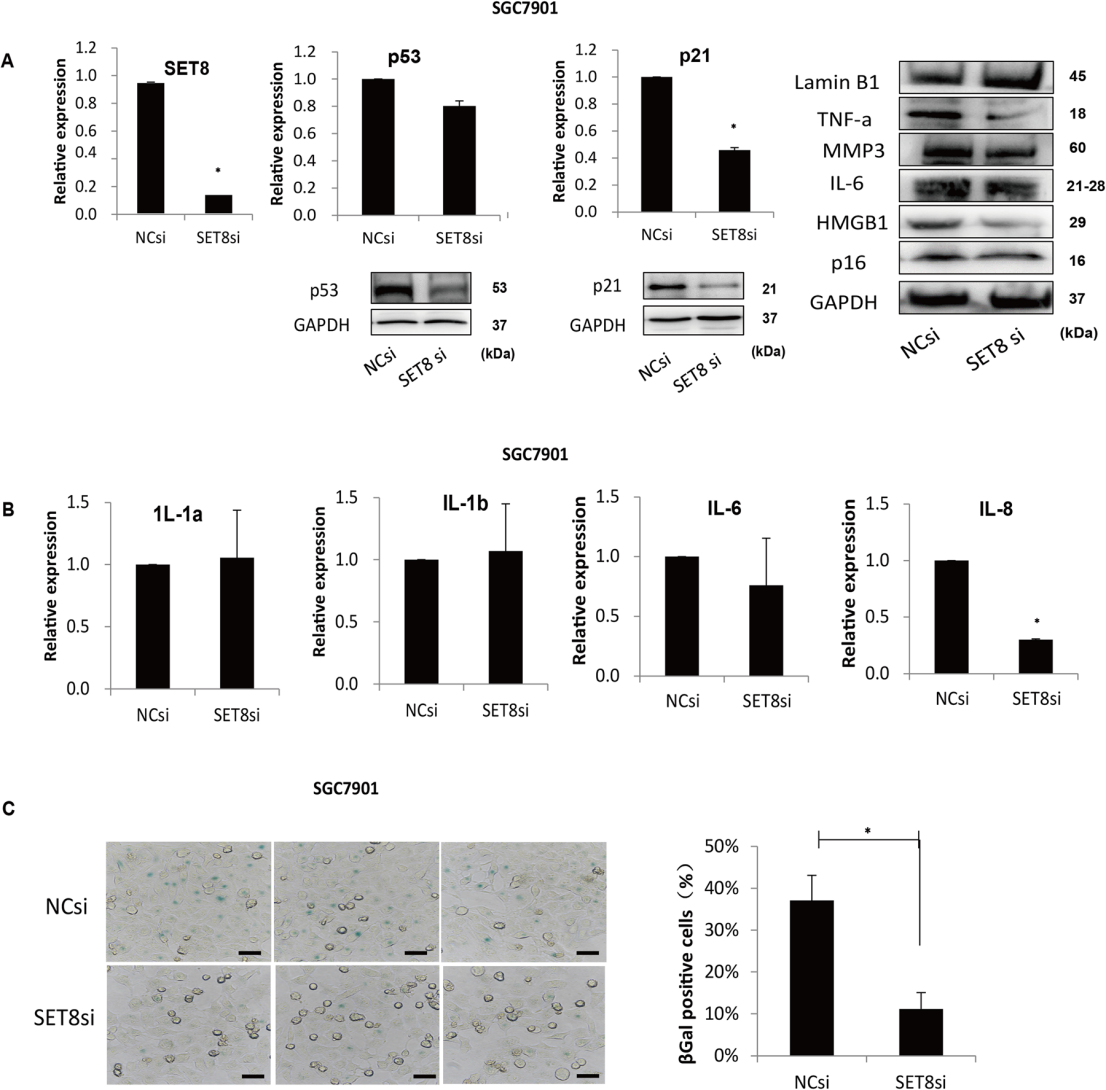
P53 and p21 are involved in the OIS induced by SET8 in GC. **A** SET8 positively regulated p53/p21 mRNA and protein levels (left). Senescence markers activated by SET8 were determined by western blot (right). **B** Senescence-associated secretory factors were detected by qRT-PCR. **C** Assays of SA-β-Gal staining. The bar graph at the bottom shows the proportion of SA-β-Gal-positive cells. SET8 regulated senescence was indicated by the expression of senescence markers. *P < 0.05. NC, negative control; si, siRNA.

Several studies have also shown that SET8 was involved in OIS^9^, wherein the acute expression of an oncogene led to the growth arrest phenotype. Moreover, almost all OIS inducers triggered activation of p53 and its transcriptional target p21^10^. In our study, we found that decreased expression of SET8 resulted in a low level of p53 and p21 in SGC7901 (Fig. 3A left). Immune blot indicated that attenuated expression of SET8 prevented the expression of SASP, including HMGB1, IL-6, TNF-alpha, MMP3, and p16. Moreover, the expression of Lamin B1, which is frequently less expressed in senescent cells, was inversely related to the expression of SET8 (Fig. 3A, right). Consequently, mRNA levels of SASP factors, including IL-1a, IL-1b, IL-6, and IL-8, were examined by qRT-PCR. The results show that IL-6 and IL-8 were notably downregulated by knockdown of SET8 in SGC7901 (Fig. 3B). To further ascertain whether SET8-induced senescence in GC, SA-β-Gal staining was performed. It was found that SA-β-Gal positivity also varied with senescent state of cells. Upon inhibition of SET8, cells positive for SA-β-Gal were decreased significantly (Fig. 3C). In summary, these data show that p53 may play a role in the progress of GC by assisting SET8 to induce senescence.

### miR-192/215 targeting SET8 accelerated the progression of GC

**Fig. 4:**
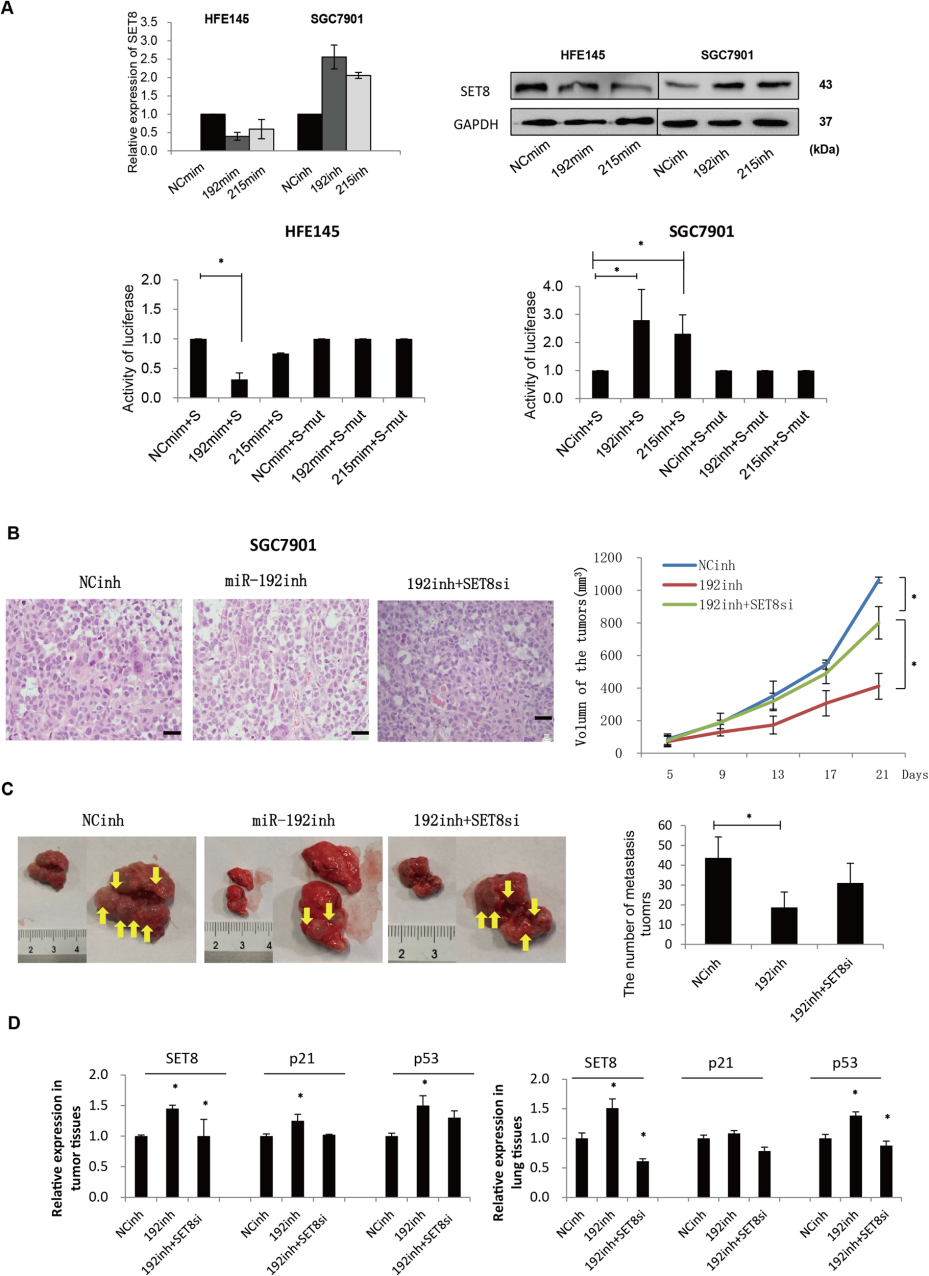
miR-192-SET8 axis inhibits p53/p21 to regulate GC cell proliferation and metastasis. **A** Up: detection of mRNA and protein levels of SET8 regulated by miR-192/215. Transfection of miR-192/215 inhibitors, SET8 was significantly increased in SGC7901 and HFE145. Conversely, SET8 was significantly downregulated in cells treated with miR-192/215 mimics. Down: results of the luciferase reporter assays. Transfection of miR-192/215 mimics significantly downregulated the firefly luciferase activity of SET8-3′-UTR-wt vector, whereas the SET8-3′-UTR-mut vector was upregulated. NC, negative control in all experiments. **B** Subcutaneous tumor xenograft assays. The growth of subcutaneous tumors was suppressed by inhibition of miR-192. Co-miR-192inh and SET8si significantly reversed this growth suppression. **C** Assays of lung metastasis of nude mice. The results suggest that the number of metastatic lesions decreased significantly after transfection of miR-192 inhibitors, whereas co-transfection of miR-192 inhibitors and SET8si led to an increase in the metastatic lesions. **D** Examination of mRNA levels of SET8, p53, and p21. In mice tissues, knockdown of SET8 was effective. Meanwhile, p53 and p21 were clearly regulated by SET8. 192, miR-192; inh, inhibitor; si, siRNA. GAPDH as a loading control; mut, mutation; S, SET8-3′-UTR-wt vector. S-mut, SET8-3′-UTR-mutant vector. *P < 0.05. Error bars represent ±SD.

Gene microarray analyses of GC cells with over- or under-expression of miR-192/215 were used to screen for the targets of miRs. SET8 was found to be one of the differentially expressed genes with twofold changes (Figure S4A and Table S2). Therefore, we focused on the miR-192/215-SET8 axis in GC. Given the existence of a putative binding site between 3′-UTR of SET8 and miR-192/215, we constructed luciferase reporter vectors containing the wild-type and mutant SET8-3′-UTR (Figure S4B). Further analysis by qRT-PCR and protein blot confirmed that the expression of SET8 was decreased by miR-192/215 mimics, and then upregulated by miR-192/215 inhibitors (Fig. 4A up), which confirmed the inverse correlation between these factors. Briefly, we generated reporter constructs by inserting the SET8-3′-UTR region (wild type) downstream of the firefly luciferase gene. The results revealed that miR-192/215 notably suppressed the luciferase activity in the wild-type SET8 reporter, but not in the mutant (Fig. 4A down). Thus, we had demonstrated that miR-192/215 directly regulated 3′-UTR of SET8 in GC.

Subcutaneous tumor xenograft assays were carried out to explore the miR-192-SET8 axis in GC. Treatment of SGC7901 cells with miR-192 inhibitors resulted in decreased xenograft growth compared with an NC group. Conversely, xenograft growth was rescued significantly in the miR-192 inhibitors/SET8si co-treated group (Fig. 4B). Tail-vein injection-based in vivo metastasis assays were also performed to assess the metastatic influence of the miR-192-SET8 axis on GC development. The results indicated that inhibition of miR-192 significantly restrained the lung metastases of nude mice. In addition, co-transfection of miR-192 inhibitors and SET8si rescued the lung metastases, although no significant difference was observed (Fig. 4C).

We found that SET8-based induction of OIS in GC was related to p53 and p21. Therefore, we measured the expression of p53 and p21 in tumor tissues, which revealed that inhibition of miR-192 led to increased expression of p53 and p21. Conversely, after co-transfection of miR-192 inhibitor and SET8si, decreased expression of p53 and p21 were observed (Fig. 4D). Therefore, these data show that the miR-192-SET8 axis could enhance the progress of GC by acting via p53.

### MiR-192/215-SET8 axis promoted GC progression by halting DDR-dependent senescence in GC

**Fig. 5:**
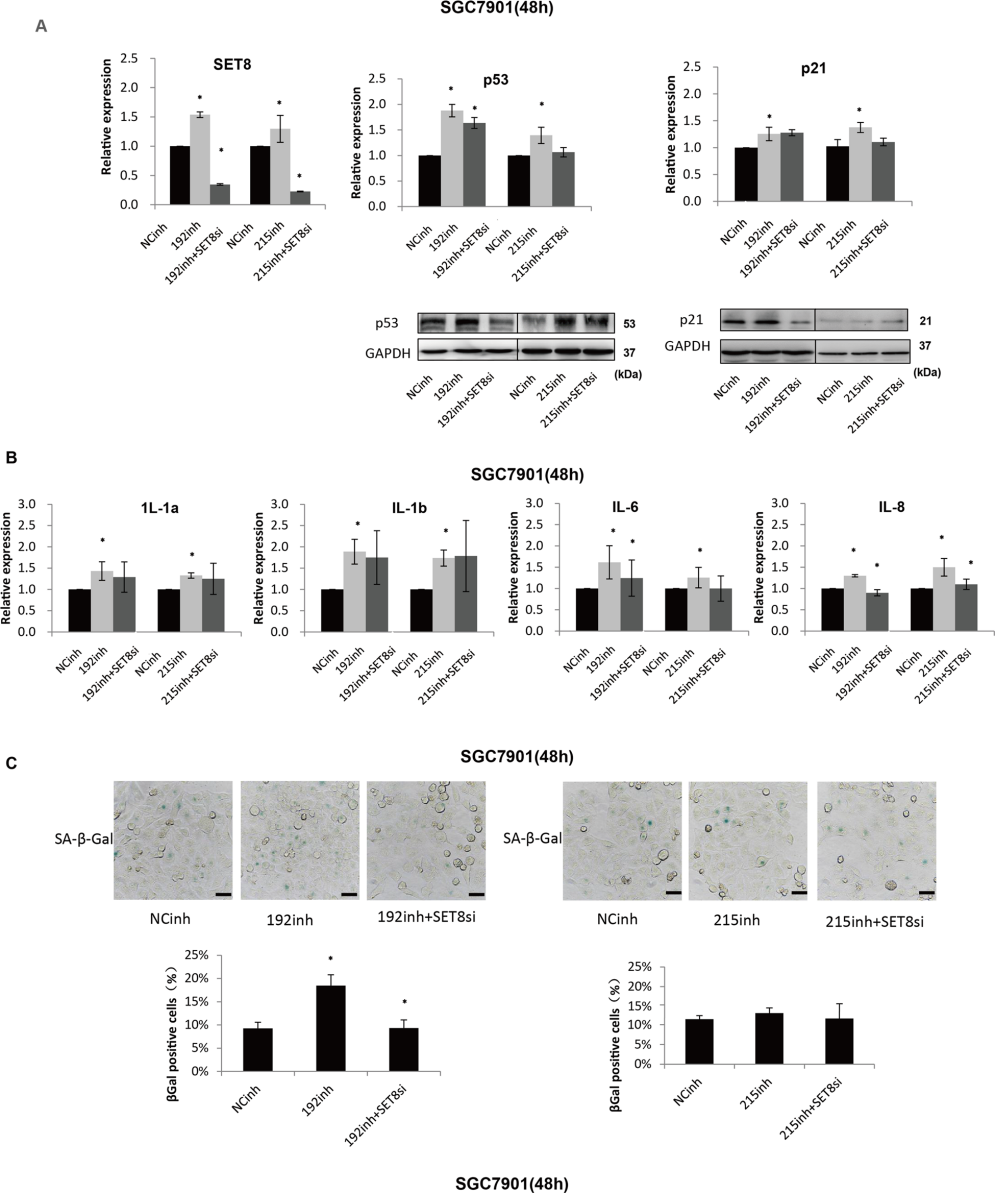
MiR-192/215-SET8 axis induces downregulation of senescence-associated secretory factors. **A**, **B** Validation of the expression of SASP, including p53, p21, IL-1a, IL-1b, IL-6 and IL-8. **C** Cells fixed and incubated with a SA-β-Gal staining solution. Cells imaged for SA-β-Gal. Percentage of SA-β-Gal-positive cells was determined for a minimum of 5 low-power magnification fields. The bar graph on the bottom shows the proportion of SA-β-Gal-positive cells (scale bar, 50 µm). 192, miR-192; 215, miR-215; inh, inhibitor; si, siRNA. *P < 0.05.

Notably, in Fig. 5A, expression of p53 and p21 were reduced in co-transfection of miR-192/215 inhibitors and SET8si in SGC7901, both at the level of mRNA and protein. It was consistent with the result of the xenograft assays. In addition, qRT-PCR of known SASP factors confirmed that inhibition of miR-192/215 significantly enhanced the expression of Il-1a, IL-1b, IL-6, and IL-8 in SGC7901 after transfection of miR-192/215 inhibitors for 48 h. Meanwhile, co-transfection of miR-192/215 inhibitors and SET8si led to the decrease of IL-6 and IL-8 expression (Fig. 5B). These results were consistent with the changes observed in SGC7901 cells after transfection of miR-192/215 inhibitors and or SET8si for 96 h (Figure S5). Staining with senescence marker SA-β-Gal demonstrated that inhibition of miR-192 led to significantly increased SA-β-Gal positivity, whereas positive cells of SA-β-Gal were decreased in miR-192 inhibitor/SET8si-treated cells. However, the regulation of miR-215 had little effect (Fig. 5C). As we speculated, levels of p53, p21, and p16 were changed by the variation in the levels of SET8: decreased expression of SET8 caused an immediate decrease in p53, p21, and p16 expression.

**Fig. 6:**
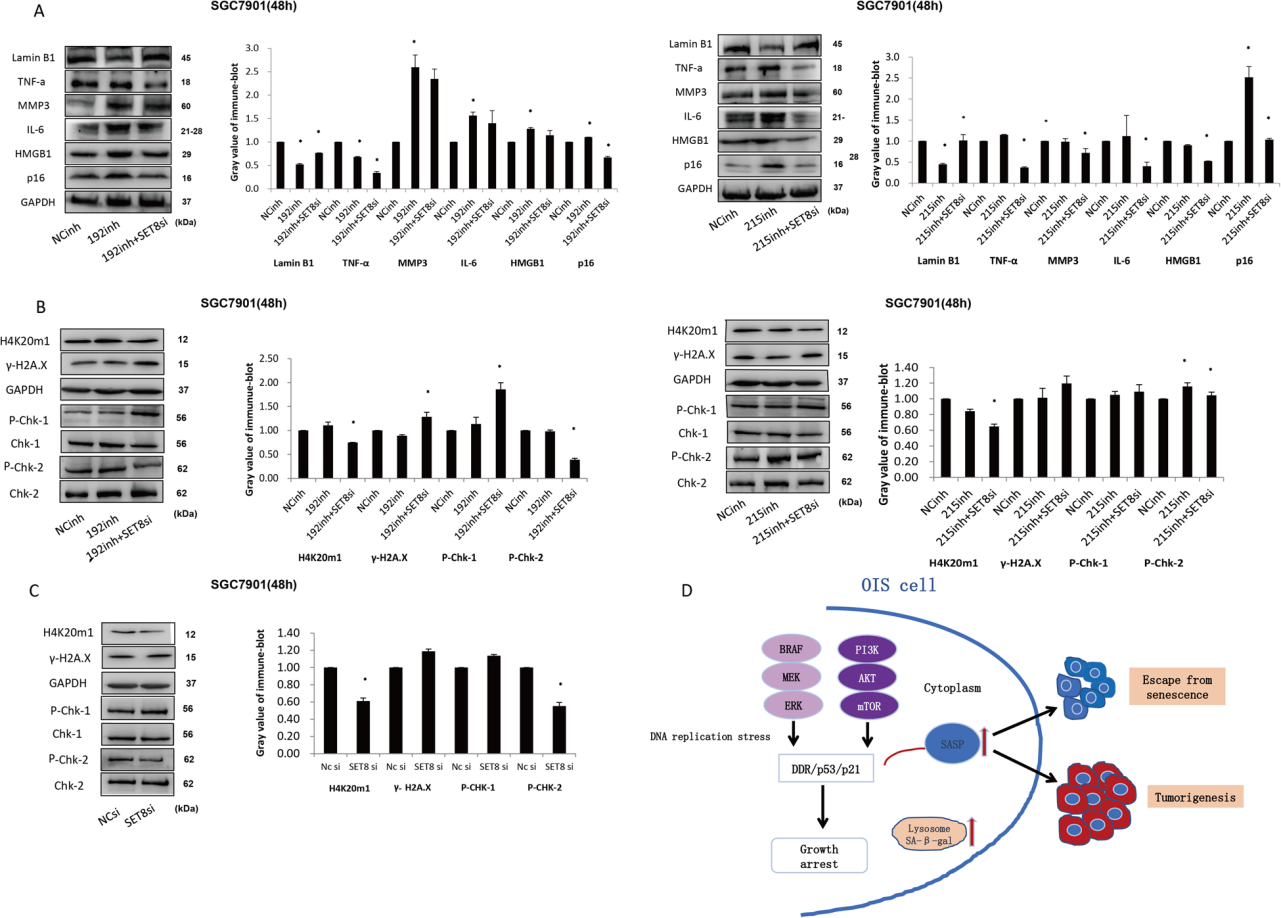
MiR-192/215-SET8 axis-mediated OIS is dependent on DDR-p53 signaling pathway. **A** Markers of senescence were detected by protein blots. **B** Protein extracts were prepared for immune-blot analysis of H4K20me1, H2A.X, Chk1, and Chk2. GAPDH serves as the internal control. **C** Alteration of SET8 uncovered targeted epigene expression during OIS in GC. **A**–**C** Gray value was measured and statistical analysis was performed. **D** Model of possible mechanism of OIS in tumors. The early phase of OIS is characterized by oncogenic signaling, and that is potentially immunosuppressive and the presence of proliferative markers. In the late phase, OIS is triggered by a DNA replication stress or DNA damage. 192, miR-192; 215, miR-215; inh, inhibitor; si, siRNA.

At the level of protein expression, SASP p16 was found to be upregulated in SGC7901 cells treated with miR-192/215 inhibitors, and downregulated in SGC7901 cells co-transfected with an miR-192/215 inhibitor and SET8si (Fig. 6A). The protein levels of SASP factors (HMGB1, IL-6, TNF-alpha, MMP3) were also examined. It was consistently found that miR-192/215 inhibitors effectively promoted the expression of these SASP factors, compared with NC. Conversely, in SGC7901 cells treated with miR-192/215 inhibitors and SET8si, the expression of these factors was significantly attenuated. Lamin B1 was present in low levels in the cells treated with miR-192/215 inhibitor, compared with NC cells, but the levels were increased in the miR-192/215 inhibitor/SET8si-treated cells (Fig. 6A). Gray value was measured, and there were some of the changes with significances. In a conclusion, SASP secreted by senescence cells are the important phenotypes of senescence. Decreased SET8 expression correlated with SASP phenotype, therefore levels of SET8 positively correlate with senescence.

DNA damage response (DDR) is the main mechanism of the initiation of OIS resulting in SASP activation and checkpoint arrests. Therefore, the expression of H4K20me1, γ-H2A.X, phospho-Chk1 (P-Chk1) and phospho-Chk2 (P-Chk2) were assayed to confirm that OIS in GC was dependent on DNA damage. In response to activation of the DDR, we used DDR agents to treat cells. CDDP was used and expression of P-Chk1, P-Chk2, formation of γ-H2AX were detected. The optimum concentration of CDDP used in different gastric cells was groped by CCK-8 assay (Figure S6A&C). We also examined the time point of the response of CDDP treatment. The results showed that expression of pChk1, pChk2, and γ-H2AX was increased at 8 and 24 h (Figure S6B&D). Moreover, we examined the changes of senescence-associated proteins. It suggested that HAMGB1, IL-6, MMP3, and p53 were increased apparently after 2 h of treatment (Figure S7). All these changes were in accordance with the result of the CDDP treatment. Moreover, we observed that the expression of H4K20me1 was upregulated by miR-192/215 inhibitors, and lowered in cells treated with miR-192/215 inhibitors and SET8si in response to senescence induction. In addition, miR-192/215 inhibitors resulted in the augment of P-Chk2, although little change in the P-Chk1. Meanwhile, co-transfection of miR-192/215 and SET8si resulted in decreased stability of H4K20me1 and P-Chk2 (Fig. 6B). In addition, treatment with SET8si alone in SGC7901, expression of H4K20me1 and P-Chk2 were downregulated significantly (Fig. 6C). However, an insignificant change in the level of γ-H2AX was observed after SET8si. This study demonstrated that SET-mediated OIS in GC was partly triggered by DDR, and miR-192/215-SET8 axis regulated GC progress was dependent on OIS.

## Discussion

In this study, we confirmed that SET8 was regulated by miR-192/215 in GC, which affected the biological properties of GC cells. It showed that SET8 was downregulated at both the mRNA and protein levels in GC tissues. Intriguingly, SET8 was found to function as a negative control of DNA damage-dependent OIS in GC, which provides mechanistic insights into a novel pathway by which SET8 may operate in tumors.

Mounting evidence indicated that SET8 was also targeted by other miRs. MiR-7 targeting SET8 suppresses the invasive potential of breast cancer cells^4^. Polymorphism at the miRs’ binding site in the 3′ UTR of SET8 is associated with the risk of several tumors^11–13^. Parchami et al.^14^ determined that genetic changes in miR-27a, miR-146a, and the miR-502’s binding site of the SET8 can be the risk factors of breast cancer^14^. Diao et al.^13^ reported that single-nucleotide polymorphisms (SNP) in the miR-502 binding site of the SET8-3′ UTR seems to influence the survival of non-Hodgkin’s lymphoma (NHL). SET8 also interacts with Twist to regulate the EMT^15^. Liu et al.^5^ identified miR-502 may suppress EMT by inhibiting SET8 in breast cancer. More importantly, studies have shown that SET8 is over-expressed in various types of cancer. For example, in hepatocellular carcinoma (HCC), non-small cell lung carcinoma (NSCLC), bladder cancer, chronic myelogenous leukemia, and NHL^13,16,17^, SET8 functions largely as an oncogene. In esophageal cancers, SET8 mediated by miR-502 modifies the outcome by inhibiting proliferation and invasion and by promoting the apoptosis of tumor cells^18^. In breast cancer, SET8 is involved in EMT^15^. In sum, these studies have highlighted the key role of SET8 in tumorigenesis and its function as an oncogene, primarily associated with cancer metastasis.

However, in this study, expression of SET8 was significantly reduced in GC. Among 48 paired specimens, almost 36 pairs showed low expression of SET8 mRNA in cancer *vs*. normal tissues. By IHC, tissue-array analysis revealed that SET8 was reduced in GC tissues and located in the cell nuclei. The patient samples of GC used to measure SET8 levels showed that many tumor samples had lower SET8 levels. We seek to establish miR-192/215 downregulates SET8 levels. But it did not show significance in the GC samples. This may be due to inadequate grading of tumors or inadequate samples. We speculate that SET8 may be a potential negative regulator protein in GC. To the best of our knowledge, only one study has examined the expression of SET8 in GC. Thus, Shi et al.^19^ showed by IHC of 100 GC-tissue samples that SET8 was most highly expressed in GC tissues. Our findings were the opposite, meaning that the function of SET8 in GC is controversial. To resolve this dispute, we searched for possible explanations.

Fortunately, previous studies have discussed the possibility of SET8 in OIS^9^. OIS is a critical tumor-suppressor mechanism, which prevents hyper-proliferation and transformation of cells. Currently, it regarded that OIS is triggered by DNA replication stress and a concomitant activation of DDR, which resulted in cell-cycle checkpoint arrests and the SASP activation (Fig. 6D, adapted from Zhu et al.^20^). Thus, OIS is a form of replication-independent senescence that can be induced prematurely in young cells by activation of oncogenes. It can be identified by senescence biomarkers such as SA-β-Gal and by the secretion of proteins such as cytokines, growth factors, and proteases. In support of this notion, several groups found that OIS can also be induced by other oncogenes such as Braf^V600E^, AKT, cyclin E^21^, and Ha-Ras^22^. Garnett et al.^23^ showed that in a mouse model of lung cancer, before OIS, p53 loss was permissive for the transition from lung adenoma to adenocarcinoma. In contrast, after OIS was established via induction by Braf^V600E^, p53 loss led to senescence and the failure of disease progression. Also involved is the PI3K/AKT/mTOR pathway, which is frequently activated in human cancer. Thus, whereas the myristoylated form of AKT induced OIS in human endothelial cells via a p53/p21-dependent pathway^24^, almost all OIS inducers were found to trigger activation of p53 and its transcriptional target p21 and/or increase the expression of p16^22^.

Importantly, emerging evidence suggests that SET8 is also involved in OIS. Wang et al.^9^ demonstrated that SET8 induced senescence via CRL4^Cdt2^ or SCFb-TRCP and led to growth arrest via an OIS-based mechanism. These workers found that SCFb-TRCP earmarked SET8 for ubiquitination and degradation in a casein kinase I-dependent manner and was activated by DNA-damaging agents. Biologically, it was determined that both CRL4^Cdt2^- and SCFb-TRCP-mediated pathways contribute to ultraviolet-induced SET8 degradation to control cell-cycle progression, governing the onset of DNA damage-induced checkpoints^9^. In addition, Benamar et al.^25^ demonstrated that CDT2 induced SET8- and p21-dependent DNA re-replication and senescence to inhibit growth in a panel of melanoma cell lines. Moreover, degradation of the CRL4-CDT2-SET8/p21 axis is the primary target of inhibition by pevonedistat in melanoma. Therefore, SET8 is both necessary and sufficient to promote re-replication program^25^. Given this presumably tumor-suppressive nature, senescence can be viewed as a barrier to cancer and therefore constitutes a potential target for a therapeutic regimen. Therefore, we speculated that SET8 induced replication-independent senescence in GC.

Almost all OIS inducers triggered the activation of p53 and induced the expression of its transcriptional target p21^22^. Accumulating documents proved p53 was related with the progression and treatment of GC. It was reported that HDAC4 regulated by miR-140 reduced cell proliferation via the p53-p73/BIK pathway in GC^26^. Meanwhile, in colon cancer, osteosarcoma and lung adenocarcinoma, miR-192/215 and p53 were found to interact^27–29^. Song et al.^28^ showed that miR-192 regulates cellular proliferation via the p53-miRNA circuit in colon carcinoma. Our study showed that inhibition of miR-192/215 induced elevated expression of p53 and p21 in GC cells. In contrast, co-transfection of miR-192/215 inhibitors and SET8si into cells led to attenuated levels of p53 and p21. Meanwhile, changes in levels of secreted protein IL-6 and IL-8 were consistent with levels of p53 and p21, although no changes were seen in the levels of IL-1a and IL-1b. Furthermore, the staining of SA-β-Gal and senescence-associated proteins was positively correlated with the level of SET8. Thus, according to these various biological functions of SET8, it was regarded as a potential oncogene, but one that induced senescence irreversibly in initiation or progression of GC, acting via p53. Without a doubt, further investigations will be required to decipher how SET8 functions oncogenically and interacts with p53 to induce senescence in GC.

OIS is a tumor-suppressing defense mechanism during tumorigenesis that inhibits oncogenic transformation in multiple human tumor types and serves as an initial barrier to cancer development in vivo^10,21^. The exact mechanism by which dysregulated SET8 protein promotes re-replication and senescence in GC remains unclear. However, another study found that these functioned as tumorigenesis barriers, involving DNA replication stress and OIS, and that these two barriers were tightly associated^14,20,26^. Some oncogenes induce OIS via DNA damage responses by hyper-replication of DNA caused by sustained oncogenic signals^12,13^. SET8 is a histone H4-lysine 20 methyltransferase required for normal cell proliferation. Inhibition of SET8 methyltransferase activity causes replicative stress. Therefore, histone H4K20 methylation may be critical for this activity^30,31^. In addition, Chk1 and Chk2 are early markers of the DNA damage response.

We found that SET8si immediately restrained H4K20m1, consistent with a previous report^32^. The inhibition of miR-192/215 increased the expression of H4K20m1, and the co-transfection of miR-192/215 inhibitors and SET8si caused the downregulation of H4K20m1. The levels of the DNA damage marker P-Chk2 were also attenuated by SET8si in our assays, which was consistent with the previous report^25^. Co-transfections rescued the expression successfully. Once DNA damage occurred, it activated the DNA damage checkpoint and led to apoptosis^12^. Chk2 coordinates the repair for S-phase entry (G1 checkpoint) via phosphorylation of p53, which transactivates p21. In conclusion, SET8-induced OIS is potentially associated with DNA damage.

**Fig. 7:**
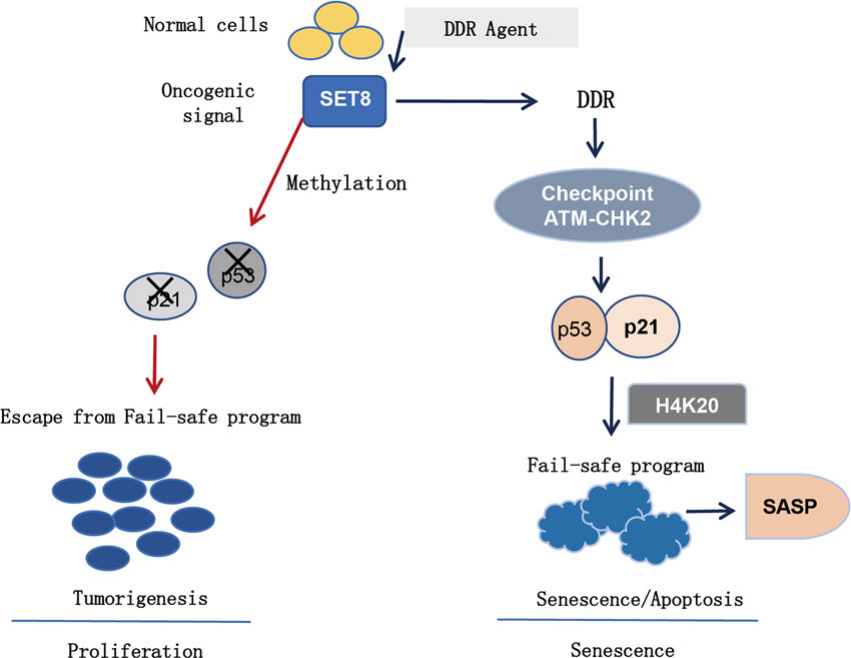
Proposed model of SET8/P53-senescence axis hypothesis. Physiological levels of SET8 in GC led to the activation of senescence that enhanced p53 expression, and promote the fail-safe program. SASP increased in the senescence cells.

Taken together, our data are consistent with the following model. Primarily, SET8 is an oncogene, which suppressed p53/p21 pathway by methylation, tending to transform normal cells to pre-neoplastic cells. However, senescence functions as a barrier to promote fail-safe programs to protect the normal cells from transformation, operating via a process called OIS. In this study, we found DDR mechanism participated in the initiation of OIS in GC. The major tumor-suppressor pathways are activated: p53/p21 (Fig. 7). As a result, in combination with miR-192/215, the regulator of SET8, a regulatory axis is formed that halts the expression of p53 and results in the inhibition of cellular senescence. Ultimately, this results in pre-neoplastic cells becoming cancer cells*.*

In this study, we revealed the existence of a new mechanism in GC that involves SET8 regulation by miR-192/215. Thus, the miR-192/215-SET8 axis promotes the initiation and progress of GC in response to DNA damage and the occurrence of senescence. Because the expression levels of SET8 in tumor tissues are significantly low, it may constitute an effective research target for cancer therapy. Therefore, further functional analyses should explore the SET8-dependent OIS pathway as a possible research target in various types of cancer.

## Supplementary information

Supplementary figure 1

Supplementary figure 2

Supplementary figure 3

Supplementary figure 4

Supplementary figure 5

Supplementary figure 6

Supplementary figure 7

Supplementary Table 1

Supplementary Table 2

## References

[1] Jin Z (2011). MicroRNA-192 and -215 are upregulated in human gastric cancer in vivo and suppress ALCAM expression in vitro. Oncogene.

[2] Zhang X (2018). Inhibition of the miR-192/215-Rab11-FIP2 axis suppresses human gastric cancer progression. Cell Death Dis..

[3] Couture JF, Collazo E, Brunzelle JS, Trievel RC (2005). Structural and functional analysis of SET8, a histone H4 Lys-20 methyltransferase. Genes Dev..

[4] Yu N (2013). microRNA-7 suppresses the invasive potential of breast cancer cells and sensitizes cells to DNA damages by targeting histone methyltransferase SET8. J. Biol. Chem..

[5] Liu B (2016). MiR-502/SET8 regulatory circuit in pathobiology of breast cancer. Cancer Lett..

[6] Guo F (2014). Post-transcriptional regulatory network of epithelial-to-mesenchymal and mesenchymal-to-epithelial transitions. J. Hematol. Oncol..

[7] Peng Y (2017). MiRNA-194 activates the Wnt/beta-catenin signaling pathway in gastric cancer by targeting the negative Wnt regulator, SUFU. Cancer Lett..

[8] Wang C, Guo Z, Wu C, Li Y, Kang S (2012). A polymorphism at the miR-502 binding site in the 3′ untranslated region of the SET8 gene is associated with the risk of epithelial ovarian cancer. Cancer Genet..

[9] Wang Z (2015). SCF(beta-TRCP) promotes cell growth by targeting PR-Set7/Set8 for degradation. Nat. Commun..

[10] Xu Y, Li N, Xiang R, Sun P (2014). Emerging roles of the p38 MAPK and PI3K/AKT/mTOR pathways in oncogene-induced senescence. Trends Biochem. Sci..

[11] Guo Z (2012). A polymorphism at the miR-502 binding site in the 3′-untranslated region of the histone methyltransferase SET8 is associated with hepatocellular carcinoma outcome. Int. J. Cancer.

[12] Xu JS (2016). Polymorphism at the miR-502 binding site in the 3′ untranslated region of SET8 gene is associated with the risk of clear cell renal cell carcinoma. Zhonghua zhong liu za zhi [Chinese journal of oncology].

[13] Diao L (2014). Prognostic value of microRNA 502 binding site SNP in the 3′-untranslated region of the SET8 gene in patients with non-Hodgkin’s lymphoma. Tumori.

[14] Parchami Barjui S, Reiisi S, Ebrahimi SO, Shekari B (2017). Study of correlation between genetic variants in three microRNA genes (hsa-miR-146a, hsa-miR-502 binding site, hsa-miR-27a) and breast cancer risk. Curr. Res. Transl. Med..

[15] Yang F (2012). SET8 promotes epithelial-mesenchymal transition and confers TWIST dual transcriptional activities. EMBO J..

[16] Chen X (2019). Monomethyltransferase SET8 facilitates hepatocellular carcinoma growth by enhancing aerobic glycolysis. Cell Death Dis..

[17] Takawa M (2012). Histone lysine methyltransferase SETD8 promotes carcinogenesis by deregulating PCNA expression. Cancer Res..

[18] Wang C, Wu J, Zhao Y, Guo Z (2016). miR-502 medaited histone methyltransferase SET8 expression is associated with outcome of esophageal squamous cell carcinoma. Sci. Rep..

[19] Shi XL, Guo ZJ, Wang XL, Liu XL, Shi GF (2015). SET8 expression is associated with overall survival in gastric cancer. Genet. Mol. Res.: GMR.

[20] Zhu H (2020). Oncogene-induced senescence: from biology to therapy. Mechanisms Ageing Dev..

[21] Courtois-Cox S, Jones SL, Cichowski K (2008). Many roads lead to oncogene-induced senescence. Oncogene.

[22] Serrano M, Lin AW, McCurrach ME, Beach D, Lowe SW (1997). Oncogenic ras provokes premature cell senescence associated with accumulation of p53 and p16INK4a. Cell.

[23] Garnett S, Dutchak KL, McDonough RV, Dankort D (2017). p53 loss does not permit escape from Braf(V600E)-induced senescence in a mouse model of lung cancer. Oncogene.

[24] Miyauchi H (2004). Akt negatively regulates the in vitro lifespan of human endothelial cells via a p53/p21-dependent pathway. EMBO J..

[25] Benamar M (2016). Inactivation of the CRL4-CDT2-SET8/p21 ubiquitylation and degradation axis underlies the therapeutic efficacy of pevonedistat in melanoma. EBioMedicine.

[26] Spaety, M. E. et al. HDAC4 levels control sensibility toward cisplatin in gastric cancer via the p53-p73/BIK pathway. *Cancers* **11**, 10.3390/cancers11111747 (2019). 10.3390/cancers11111747 PMC6896094 31703394

[27] Braun CJ (2008). p53-Responsive micrornas 192 and 215 are capable of inducing cell cycle arrest. Cancer Res..

[28] Song B (2008). miR-192 Regulates dihydrofolate reductase and cellular proliferation through the p53-microRNA circuit. Clin. Cancer Res..

[29] Georges SA (2008). Coordinated regulation of cell cycle transcripts by p53-Inducible microRNAs, miR-192 and miR-215. Cancer Res..

[30] Tardat M, Murr R, Herceg Z, Sardet C, Julien E (2007). PR-Set7-dependent lysine methylation ensures genome replication and stability through S phase. J. Cell Biol..

[31] Abbas T (2010). CRL4(Cdt2) regulates cell proliferation and histone gene expression by targeting PR-Set7/Set8 for degradation. Mol. Cell.

[32] Beck DB, Oda H, Shen SS, Reinberg D (2012). PR-Set7 and H4K20me1: at the crossroads of genome integrity, cell cycle, chromosome condensation, and transcription. Genes Dev..

