# Supplementary figures and images for: Histone methyltransferase SET8 is regulated by miR-192/215 and induces oncogene-induced senescence via p53-dependent DNA damage in human gastric carcinoma cells
Source: Cell Death Dis. 2020 Oct 30;11(10):937. doi: 10.1038/s41419-020-03130-4 (PMC7599338; doi:10.1038/s41419-020-03130-4)

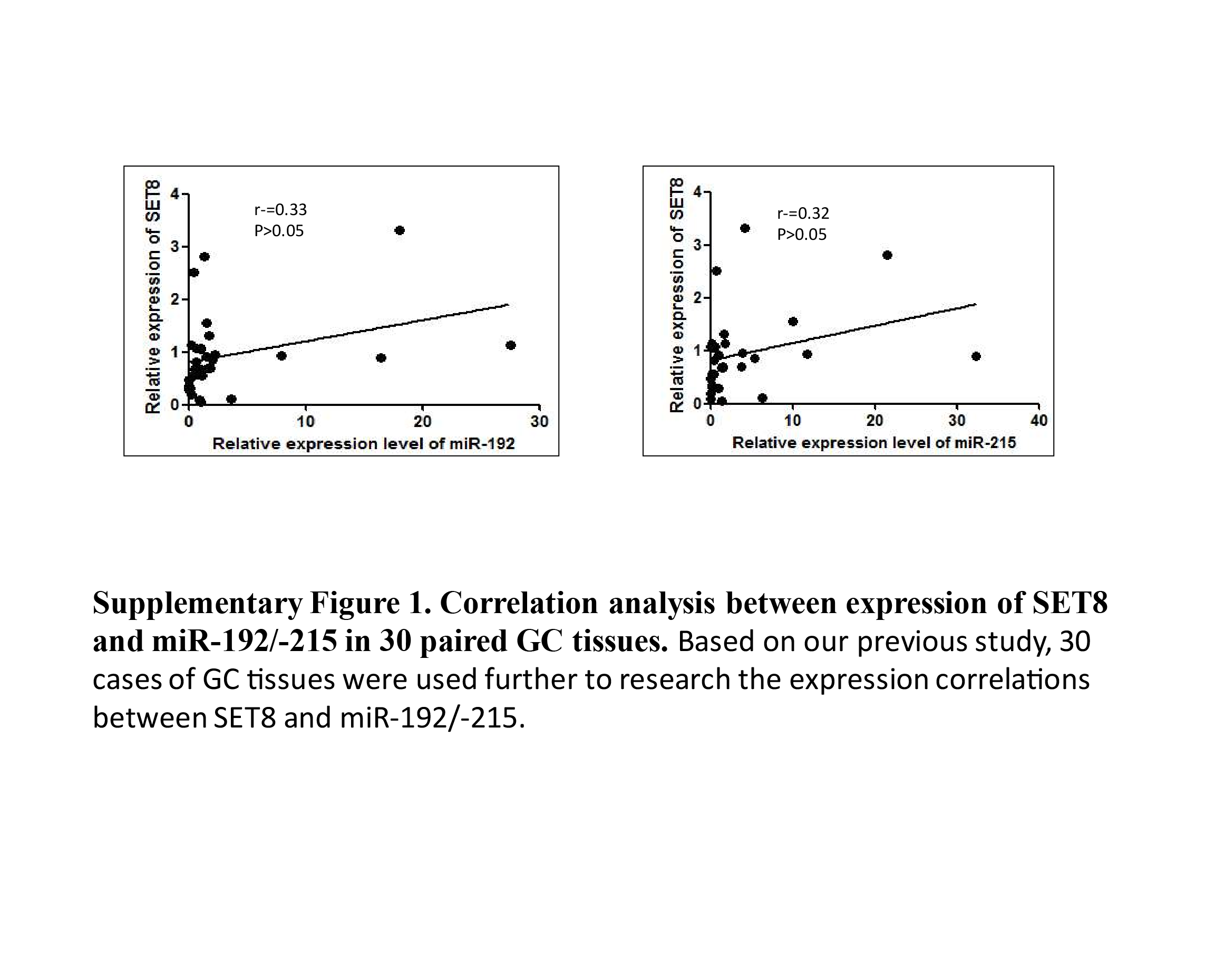

Supplement: Supplementary file 1 — Supplementary figure 1 [file 41419_2020_3130_MOESM1_ESM.png]

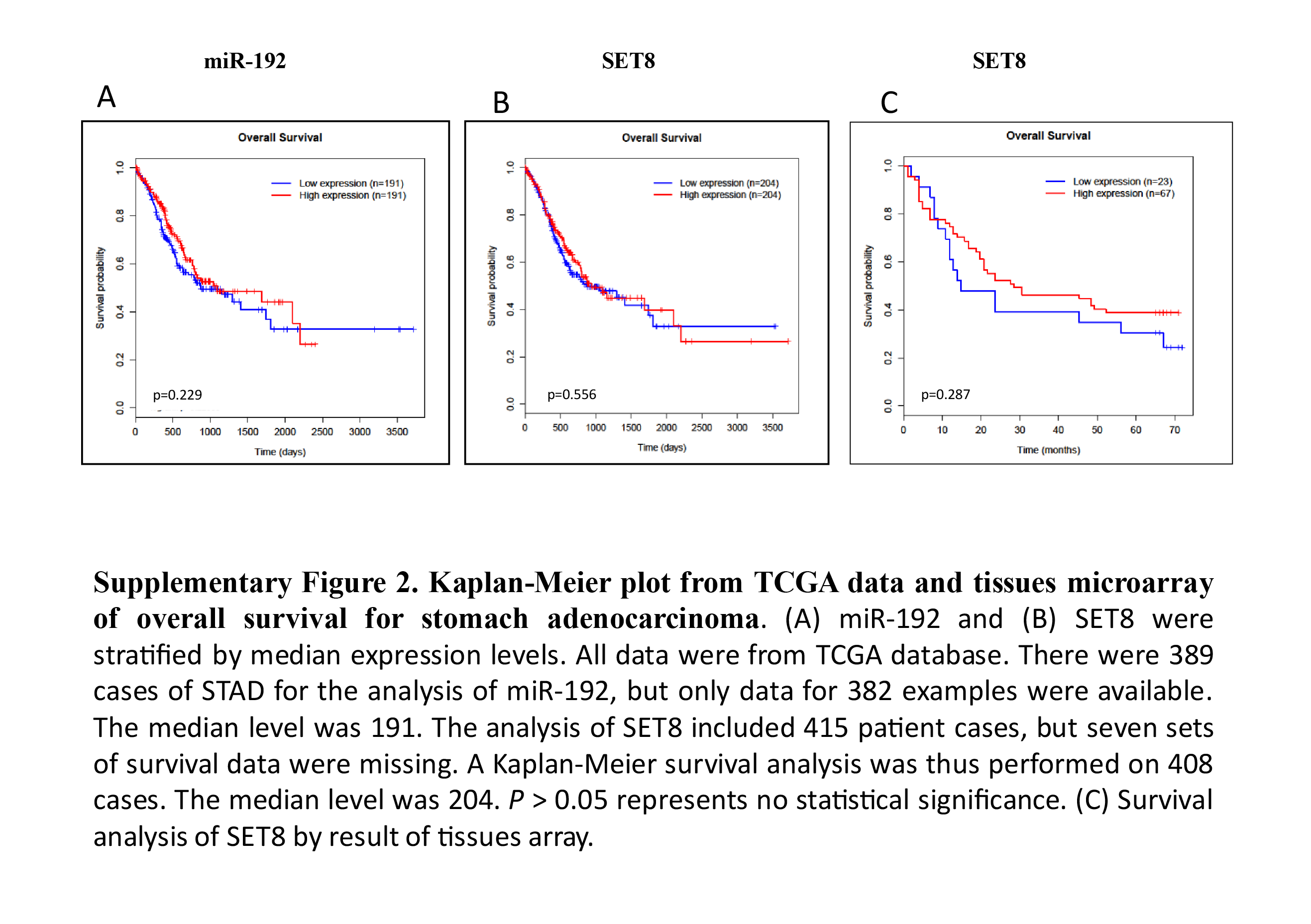

Supplement: Supplementary file 2 — Supplementary figure 2 [file 41419_2020_3130_MOESM2_ESM.png]

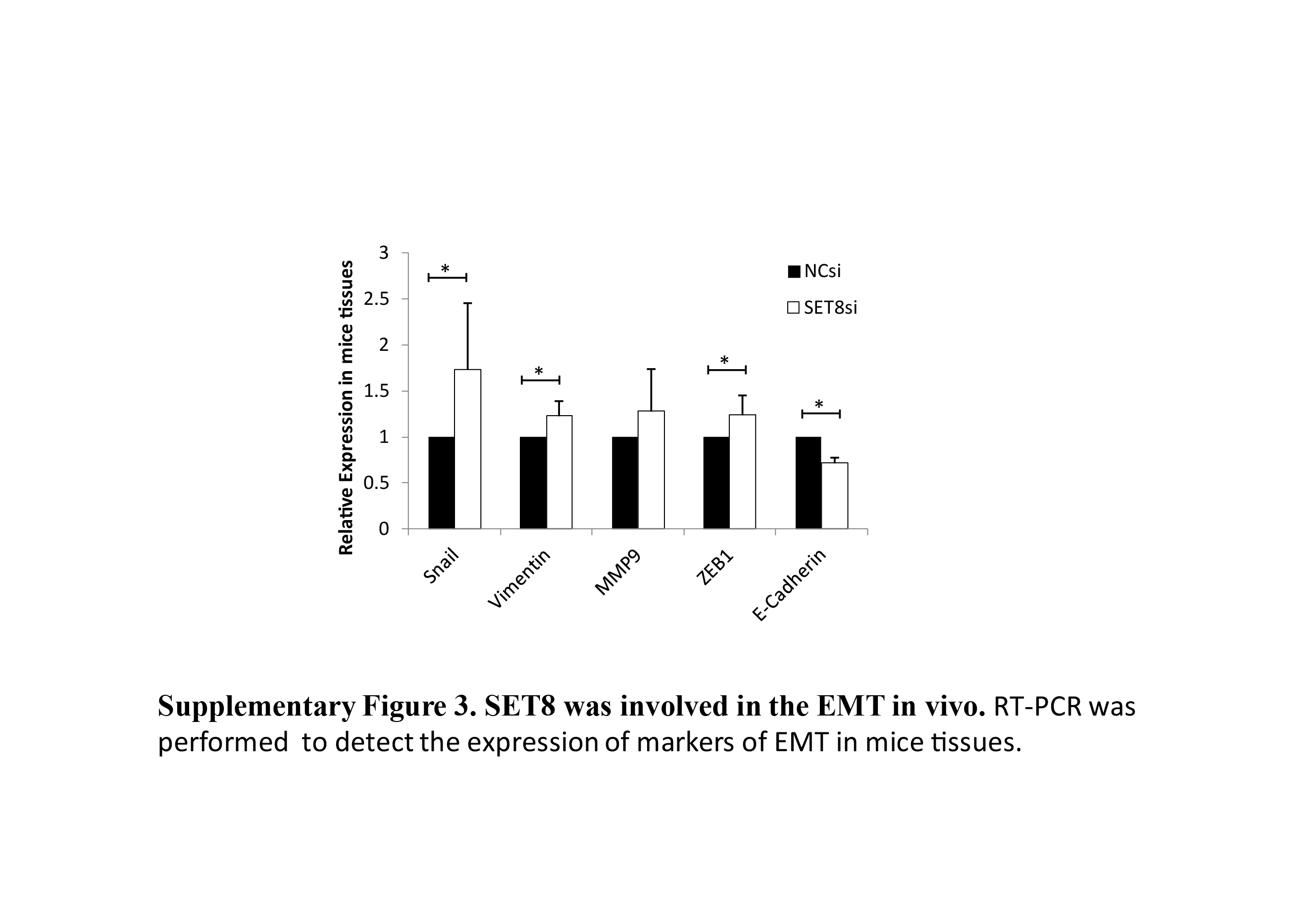

Supplement: Supplementary file 3 — Supplementary figure 3 [file 41419_2020_3130_MOESM3_ESM.png]

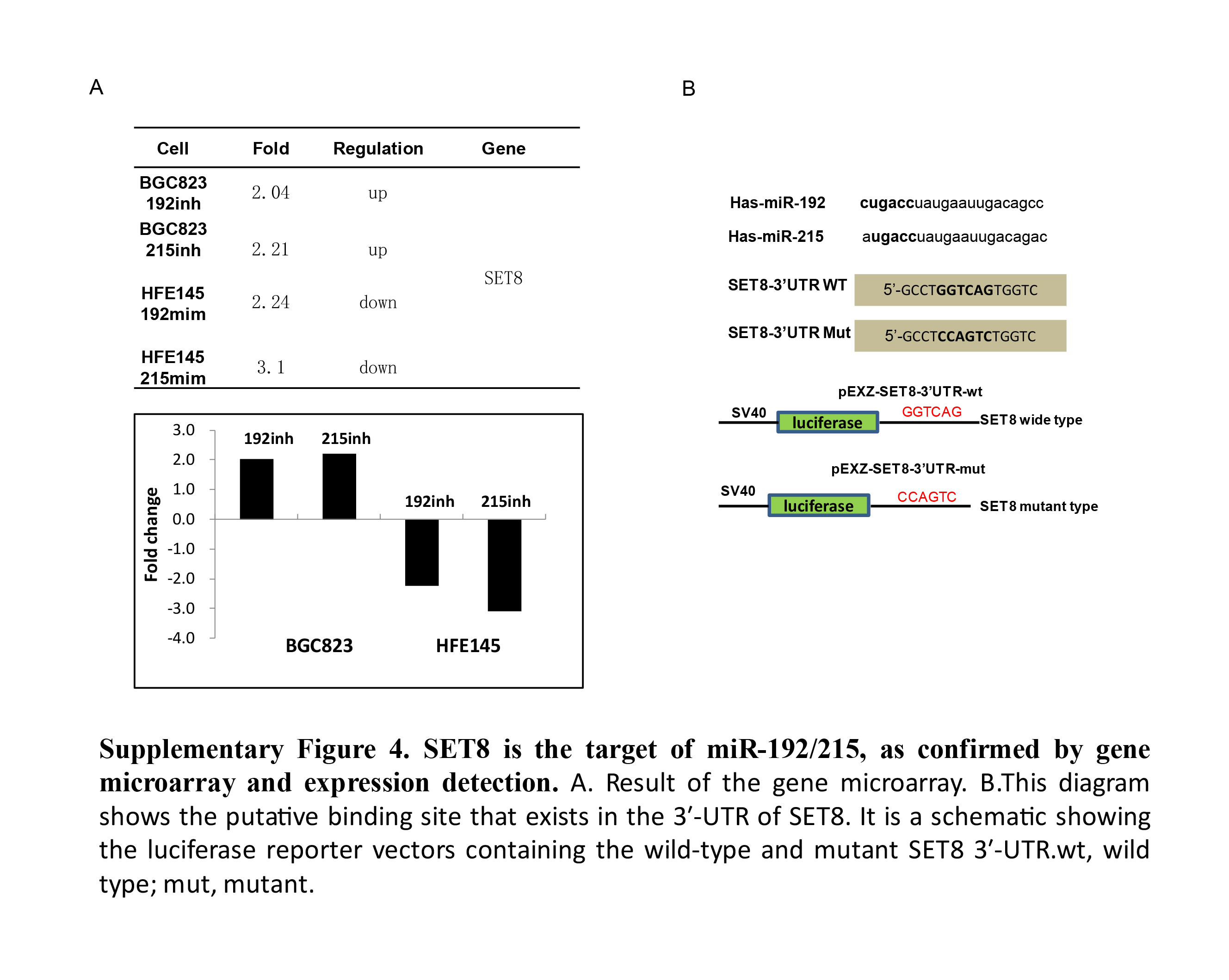

Supplement: Supplementary file 4 — Supplementary figure 4 [file 41419_2020_3130_MOESM4_ESM.png]

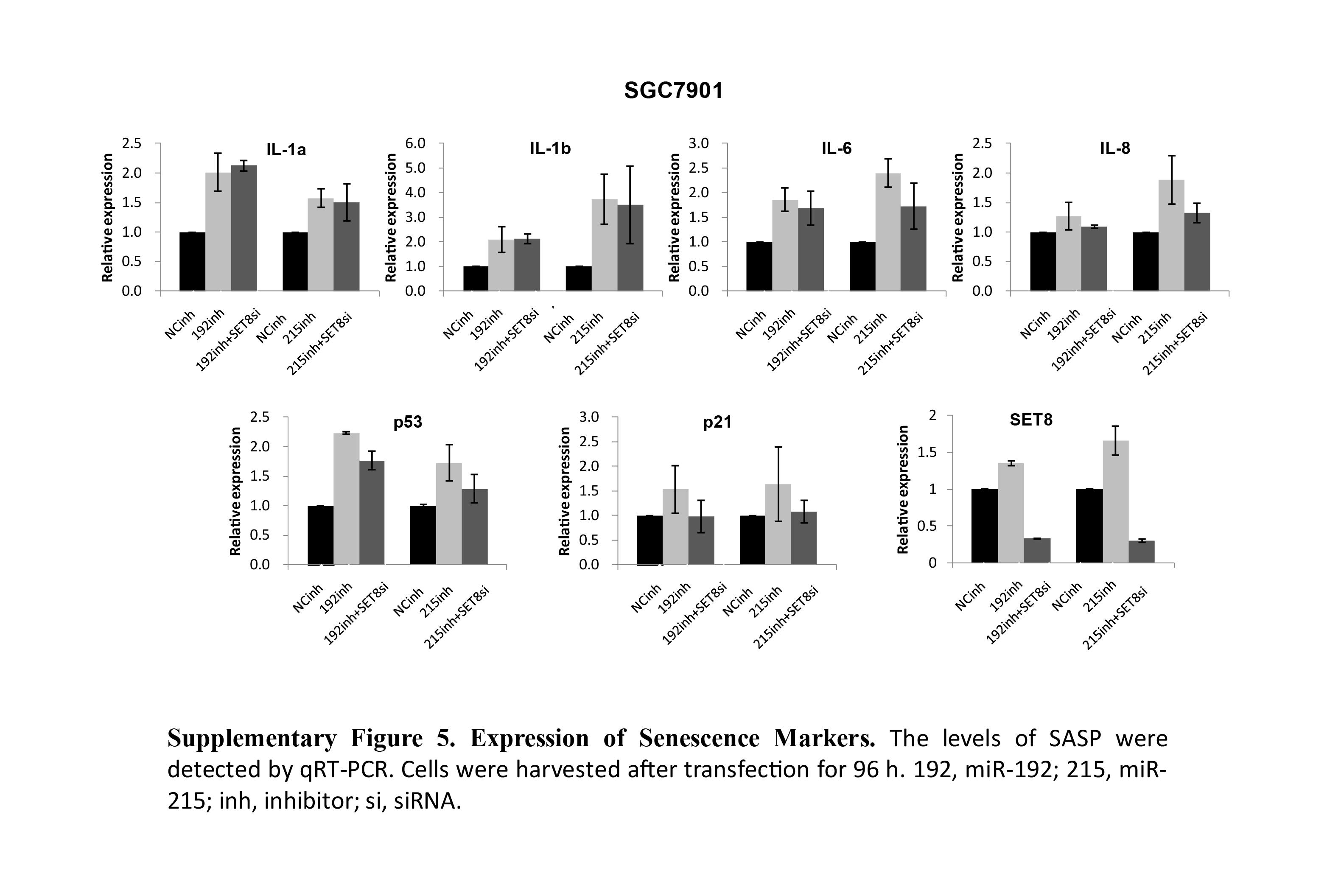

Supplement: Supplementary file 5 — Supplementary figure 5 [file 41419_2020_3130_MOESM5_ESM.png]

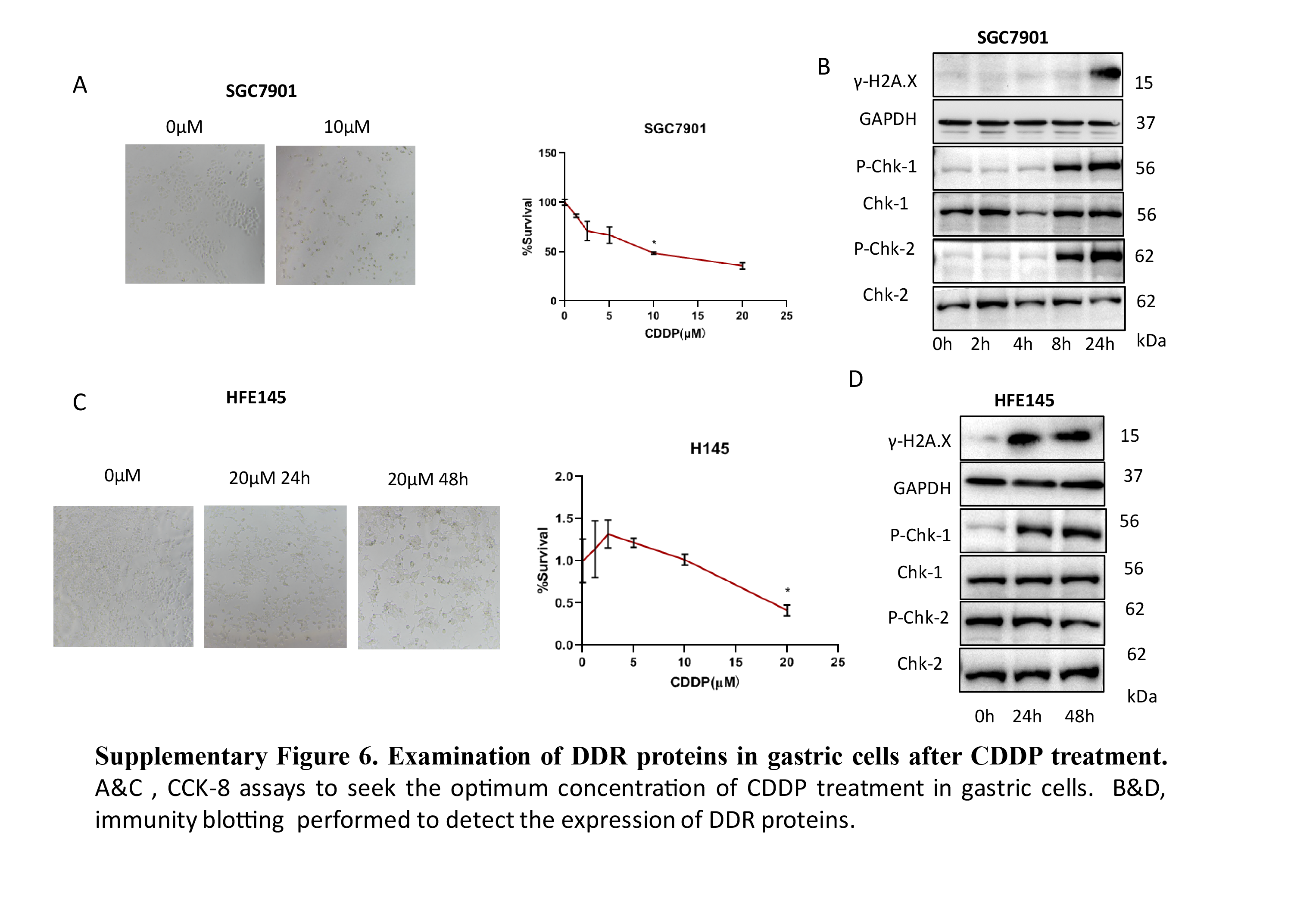

Supplement: Supplementary file 6 — Supplementary figure 6 [file 41419_2020_3130_MOESM6_ESM.png]

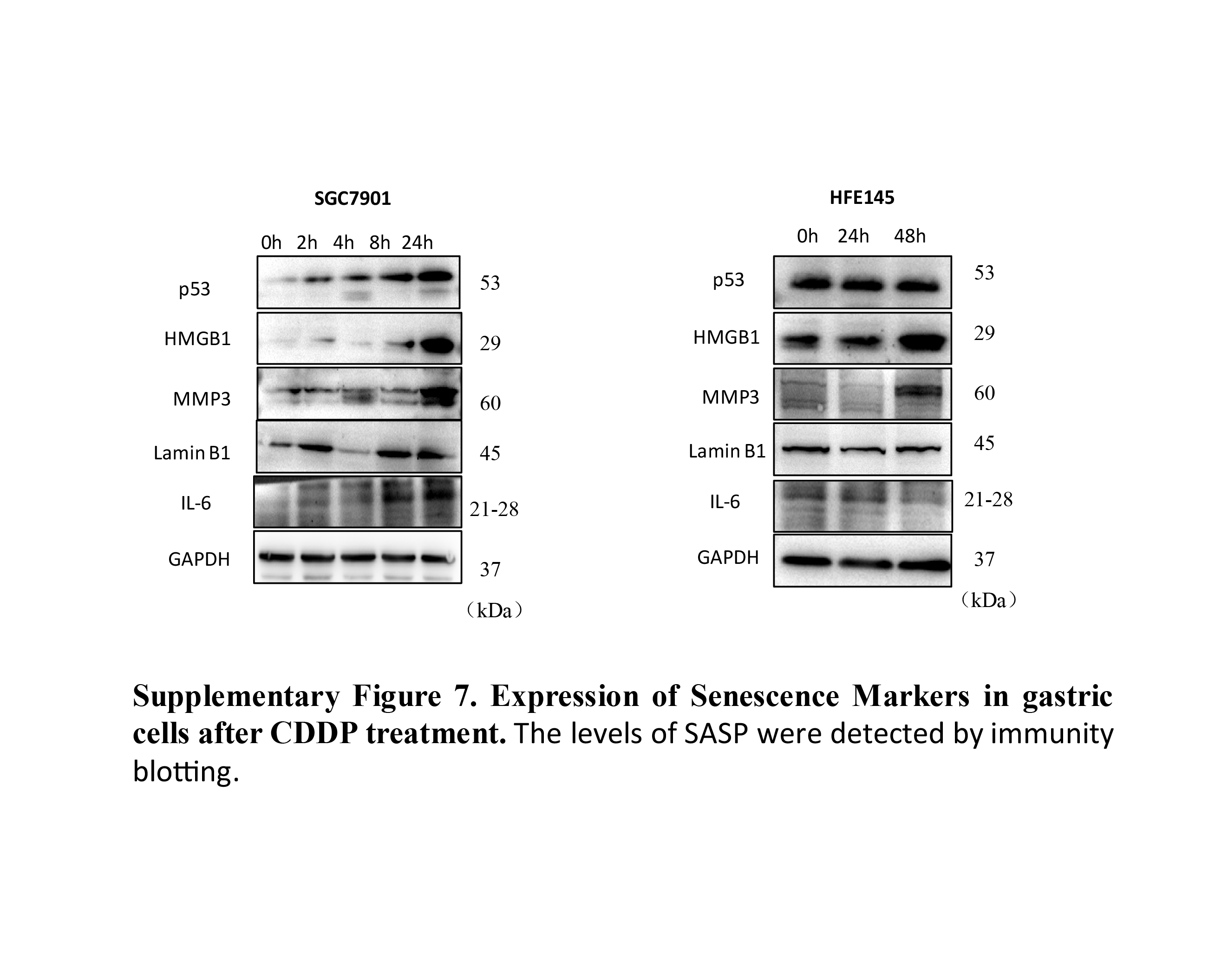

Supplement: Supplementary file 7 — Supplementary figure 7 [file 41419_2020_3130_MOESM7_ESM.png]
